# Pharmacokinetics of *Naja sumatrana* (Equatorial Spitting Cobra) Venom and Its Major Toxins in Experimentally Envenomed Rabbits

**DOI:** 10.1371/journal.pntd.0002890

**Published:** 2014-06-05

**Authors:** Michelle Khai Khun Yap, Nget Hong Tan, Si Mui Sim, Shin Yee Fung, Choo Hock Tan

**Affiliations:** 1 CENAR and Department of Molecular Medicine, Faculty of Medicine, University of Malaya, Kuala Lumpur, Malaysia; 2 Department of Pharmacology, Faculty of Medicine, University of Malaya, Kuala Lumpur, Malaysia; Universidad de Costa Rica, Costa Rica

## Abstract

**Background:**

The optimization of snakebite management and the use of antivenom depend greatly on the knowledge of the venom's composition as well as its pharmacokinetics. To date, however, pharmacokinetic reports on cobra venoms and their toxins are still relatively limited. In the present study, we investigated the pharmacokinetics of *Naja sumatrana* (Equatorial spitting cobra) venom and its major toxins (phospholipase A_2_, neurotoxin and cardiotoxin), following intravenous and intramuscular administration into rabbits.

**Principal findings:**

The serum antigen concentration-time profile of the *N. sumatrana* venom and its major toxins injected intravenously fitted a two-compartment model of pharmacokinetics. The systemic clearance (91.3 ml/h), terminal phase half-life (13.6 h) and systemic bioavailability (41.9%) of *N. sumatrana* venom injected intramuscularly were similar to those of *N. sputatrix* venom determined in an earlier study. The venom neurotoxin and cardiotoxin reached their peak concentrations within 30 min following intramuscular injection, relatively faster than the phospholipase A_2_ and whole venom (T_max_ = 2 h and 1 h, respectively). Rapid absorption of the neurotoxin and cardiotoxin from the injection site into systemic circulation indicates fast onsets of action of these principal toxins that are responsible for the early systemic manifestation of envenoming. The more prominent role of the neurotoxin in *N. sumatrana* systemic envenoming is further supported by its significantly higher intramuscular bioavailability (*F_i.m._* = 81.5%) compared to that of the phospholipase A_2_ (*F_i.m._* = 68.6%) or cardiotoxin (*F_i.m._* = 45.6%). The incomplete absorption of the phospholipase A_2_ and cardiotoxin may infer the toxins' affinities for tissues at the injection site and their pathological roles in local tissue damages through synergistic interactions.

**Conclusion/Significance:**

Our results suggest that the venom neurotoxin is absorbed very rapidly and has the highest bioavailability following intramuscular injection, supporting its role as the principal toxin in systemic envenoming.

## Author Summary

*Naja sumatrana* is a medically important cobra species in Southeast Asia. The optimization of snakebite management and the use of antivenom depend greatly on the knowledge of the venom's composition, its biological activities, as well as its pharmacokinetics. The present study on the pharmacokinetics of *N. sumatrana* venom shows that the systemic bioavailability of this venom in experimental envenomation is similar to *N. sputatrix* venom determined in an earlier study. The neurotoxin and cardiotoxin exhibited a more rapid absorption and elimination compared to the phospholipase A_2_ and the whole venom. The venom neurotoxin produced a higher systemic bioavailability than the cardiotoxin and phospholipase A_2_, suggesting that the neurotoxin plays the major toxic role in cobra bites.

## Introduction

Snake envenomation remains a neglected tropical disease prevalent in the Southeast Asia region, including Malaysia [1], [2]. It affects not only the population in the rural area but also the suburban regions due to rapid urbanization, and the encroaching of human activities into the natural habitat of snakes [3]–[7]. In Malaysia, cobra bites appears to be one of the commonest causes of snake envenomation [4]–[6]. There are two species of common cobras in Malaysia: *Naja kaouthia* and *Naja sumatrana*, both classified as Category 1 medically important venomous snake [2]. Of these two *Naja* cobras, *N. sumatrana* is widely distributed in the Peninsula Malaysia (including Singapore), and is also known as the Equatorial spitting cobra [8], one of the venom-spitting species in Southeast Asia that are able to cause venom ophthalmia. Clinically, cobra bites produce systemic envenomation syndrome with the characteristic neuromuscular paralysis, and local toxicity manifested as severe tissue necrosis [2], [6], [9]. The characterizations of different cobra venoms, however, are necessary for the better understanding of cobra envenomation pathophysiology as the toxin compositions in cobra venoms vary from species to species [10]. Recent venom profiling with the use of ion-exchange high performance liquid chromatography has shown that the major toxins of *N. sumatrana* venom comprise high abundance of phospholipase A_2_ and three-finger toxins such as polypeptides of neurotoxins and cardiotoxins [10]. These are toxins with varied biological and physicochemical properties which make the characterizations of individual toxins warranted in order to gain better insights into the toxic effects of the whole venom. The optimization of snakebite management and the use of antivenom depend greatly on the knowledge of the venom's composition, pharmacological activities, as well as its disposition in the body (*i.e.* pharmacokinetics). The pathophysiological and pharmacological effects of snake envenomation are related to the absorption and distribution kinetics of venom toxins into the systemic circulation. Indeed, it has been reported that the serum concentrations of venom antigens in snakebite victims are well correlated with the severity of systemic and local symptoms during the course of envenomation [11]. Although there have been some studies on the pharmacokinetics of snake venoms or toxins in animals [12]–[22], the highly varied snake venom compositions, inconsistent animal models, different pharmacokinetic modelling make the convergence of the data equivocal to have the pharmacokinetic parameters generalized across all snake species. To date, even within the *Naja* genus of cobras, the pharmacokinetic studies on their venoms were limited to isolated toxins of Formosan cobra [12], [21], a few African cobra venoms and their alpha toxins [15] and *N. sputatrix* venom [22]. Information on the systemic bioavailability of cobra venoms and their toxins following envenomation is even scarcer in the literature. There is therefore a need to define the pharmacokinetic parameters of specific cobra venom and its toxins more meticulously for better clinical correlation.

In the present study, the pharmacokinetics of *N. sumatrana* venom and its three major types of toxins (neurotoxin, cardiotoxin and phospholipase A_2_) were investigated using double-sandwich ELISA. This is the first report where the pharmacokinetics of a cobra venom was investigated alongside the pharmacokinetics of all its major types of toxins. The results will make it possible to interpret the pharmacokinetics of the whole venom in the light of that of its major toxins, and to enable better understanding of the pathophysiological effects of the venom.

## Methods

### Ethical statements

All experimental animals were handled in accordance to CIOMS guidelines on animal experimentation [23]. The experimental protocol on the animal study (2013-06-07/MOL/R/FSY) was approved by the Institutional Animal Care and Use Committee, Faculty of Medicine, University of Malaya.

### Venom, reagents and separation media

The venom was a pooled sample obtained from three adult *N. sumatrana* captured in central Malaysia (Negeri Sembilan) and was supplied by Snake Valley (Seremban, Malaysia).

Resource S ion exchange column and HiTrap Protein A HP affinity column were purchased from GE Healthcare (New Jersey, USA). Goat anti-rabbit IgG-horseradish peroxidase (HRP) conjugate was obtained from Abnova, Taipei, Taiwan. Lichrosphere WP 300 C_18_ reverse-phase column cartridge was purchased from Merck, New Jersey, USA. iBlot Gel Transfer stacks and iBlot blotting system were supplied by Invitrogen. Sephadex G-25 gel beads and all other reagents were purchased from Sigma – Aldrich (St. Louis, USA) or as stated in the methods.

### Animals

The animals used in this study (New Zealand white rabbits) were supplied by Chenur Supplier (Selangor, Malaysia). The animals were housed in Laboratory Animal Centre, Faculty of Medicine, University of Malaya, and received water and food *ad libitum*.

### Purification of *N. sumatrana* toxins

The major *N. sumatrana* venom toxins (phospholipase A_2_, neurotoxin and cardiotoxin) were isolated from the venom by Resource S ion-exchange chromatography as described by Yap *et al*., 2011 [10]. The isolated phospholipase A_2_, neurotoxin and cardiotoxin (corresponds to peak 5, peak 7 and peak 8, respectively as reported in Yap *et al*., 2011 [10]) were further purified by C_18_ reverse-phase high performance liquid chromatography (HPLC) to homogeneity on 12.5% sodium dodecyl sulfate polyacrylamide gel electrophoresis (SDS-PAGE). The gel bands were subjected to in-gel tryptic digestion followed by protein identification using matrix assisted laser desorption/ionization-time of flight (MALDI-TOF/TOF) mass spectrometry, as described by Yap *et al*., 2011 [10].

### Production of IgG antibodies against *N. sumatrana* venom, venom phospholipase A_2_, neurotoxin and cardiotoxin

Pre-immune serum was collected and used as the control in ELISA. In the first immunization, *N. sumatrana* venom (10 µg) or venom toxins (neurotoxin, cardiotoxin and phospholipase A_2_, respectively at 5 µg) dissolved in PBS (pH 7.2) and mixed with an equal volume of Freund's complete adjuvant, was injected intramuscularly into the thigh of the rabbits (*n* = 3 for each group). For the subsequent immunizations, 20 µg of the venom or 10 µg of venom toxins were dissolved in PBS (pH 7.2), respectively, mixed with an equal volume of Freund's incomplete adjuvant and injected intramuscularly at multiple sites at the back of the rabbit fortnightly for 8 weeks. The immunogenicity and antibody titers of inocula were monitored using indirect ELISA as described by Yap *et al*., 2011 [10]. The rabbits were bled by cardiac puncture 9 days after the final immunization as indicated by plateauing of antibody titer on indirect ELISA.

### Purification of IgG and preparation of horseradish peroxidase (HRP) conjugate

Anti - *N. sumatrana* venom IgG and three anti-toxins IgG were isolated from rabbit sera (upon completion of immunization scheme) by Sephadex G-25 gel chromatography, followed by Protein A affinity chromatography [24]. The IgG-HRP conjugate was prepared as described by Wisdom, 1996 [25].

### Investigation of immunological cross-reactivity of *N. sumatrana* major venom toxins (phospholipase A_2_, PLA_2_; neurotoxin, NTX; cardiotoxin, CTX)

#### Indirect ELISA for investigation of immunological cross-reactivity

ELISA immunoplate was coated with 100 ng/ml of venom toxin (phospholipase A_2_, PLA_2_; neurotoxin, NTX or cardiotoxin, CTX) respectively and incubated overnight at 4°C. The plates were washed with PBS-Tween and subsequently anti-PLA_2_ IgG, anti-NTX IgG or anti-CTX IgG (dilutions 1∶200) was added and allowed to incubate at room temperature for 1 h. This was followed by incubation with goat anti-rabbit IgG horseradish peroxidase conjugate (dilutions of 1∶6000) and 100 µl of substrate o-phenylenediamine dihydrochloride (0.4 mg/ml) for 1 h. The reaction was terminated by adding 50 µl sulfuric acid (12.5%). The absorbance at 492 nm was then determined using Bio-Rad Model 690 microplate reader. The degree of cross-reactivity was expressed in percentage (%) of absorbance.

#### Double-sandwich ELISA for investigation of immunological cross-reactivity

ELISA immunoplate was coated overnight at 4°C with 100 µl of anti-PLA_2_ IgG, anti-NTX IgG, anti-CTX IgG (4 µg/ml), respectively. Plates were then incubated with 100 µl of the respective venom toxins as antigens (phospholipase A_2_, neurotoxin, cardiotoxin) at a concentration of 100 ng/ml. This was followed by incubation with 100 µl of anti-toxins IgG-HRP conjugate (1∶400) for 2 h, and 100 µl of substrate o-phenylenediamine dihydrochloride (0.4 mg/ml) was then added. The reaction was terminated after 1 h by adding 50 µl of 12.5% sulfuric acid and the absorbance at 492 nm were then determined using Bio-Rad Model 690 microplate reader. The degree of cross-reactivity was expressed in percentage (%) of absorbance.

#### SDS-PAGE and western blotting (immunoblotting)

SDS-PAGE was conducted in an electrophoresis (slab) system according to the method described by Studier, 1973 [26] and a Fermentas Spectra Multicolor Broad Range Protein Ladder broad range SDS-PAGE standard was used for calibration. Ten micrograms of venom toxins (phospholipase A_2_, neurotoxin or cardiotoxin) was electrophoresed (15% gel) under reducing condition.

The proteins on the polyacrylamide gel was transferred to a polyvinylidene fluoride (PVDF) membrane (iBlot Gel Transfer stacks, PVDF, mini, Invitrogen) using iBlot blotting system (Invitrogen). The PVDF membrane was subsequently blocked wit 2% BSA in Tris-buffered saline, Tween 20. Anti-PLA_2_ IgG, anti-NTX IgG or anti-CTX IgG (dilution of 1∶500 in TBS-Tween) was added to the PVDF membrane followed by incubation with Goat anti-rabbit IgG horseradish peroxidase conjugate (dilutions of 1∶1000) for 1 h. The chromogenic detection of the protein bands on the PVDF membrane was carried out by addition of the substrate solution (Novex HRP Chromogenic Substrate (TMB), Invitrogen).

### Determination of serum venom antigen and venom toxin antigen levels using double-sandwich ELISA

Double-sandwich ELISA was conducted as described previously [27]. It was used to monitor the serum venom antigen levels following experimental envenomation in rabbits (*n* = 3) during pharmacokinetic studies. Briefly, ELISA immunoplates were coated overnight at 4°C with optimal coating concentration for venom and venom toxins, which has been optimized as stated above in the previous section (double-sandwich ELISA for investigation of immunological cross-reactivity). This was followed by subsequent incubation with 100 µl of diluted rabbit serum samples (1∶20) collected at different time intervals, 100 µl of anti-*N. sumatrana* venom IgG-HRP conjugate and anti-toxins IgG-HRP conjugate (dilution of 1∶400) for 2 h. Substrate o-phenylenediamine dihydrochloride (0.4 mg/ml) was added for colorimetric development and the absorbance at 492 nm was then determined using Bio-Rad Model 690 microplate reader. A standard curve was constructed using varying dilutions of venom or the respective toxins in the spiked pre-envenomed sera.

### Pharmacokinetics of *N. sumatrana* venom or toxins after intravenous (*i.v.*) and intramuscular (*i.m.*) administrations

The pharmacokinetics of *N. sumatrana* venom or toxins was studied using rabbits (*n* = 3). A sub-lethal dose of the venom or toxins was administered intravenously (*i.v.*, marginal ear vein) or intramuscularly (*i.m.*, quadriceps) into rabbits. Doses administered were as follow: venom 0.5 mg/kg (*i.m.*) or 0.1 mg/kg (*i.v.*); phospholipase A_2_ 0.1 mg/kg (*i.m.*) or 0.05 mg/kg (*i.v.*); neurotoxin 0.07 mg/kg (*i.m.*) or 0.05 mg/kg (*i.v.*); cardiotoxin 0.15 mg/kg (*i.m.*) or 0.05 mg/kg (*i.v.*). Blood samples were collected from central ear artery before experimental envenomation and at specific time points (5 min, 10 min, 30 min, 1 h, 2 h, 3 h, 6 h and 24 h) after venom injection. The collected blood samples were centrifuged at 3,500 g for 20 min to obtain the sera, which were kept at -20°C until further analysis. The serum antigen concentrations were measured by double-sandwich ELISA as described above using the pre-envenomed serum sample taken from the same animal as the control for baseline reading.

A parallel series of experiments were conducted to investigate the pharmacokinetics of cardiotoxin in the whole venom when *N. sumatrana* venom was injected intravenously or intramuscularly into the rabbits (*n* = 3). The serum concentrations of cardiotoxin (in the whole venom) at specified sampling times were estimated using anti-CTX IgG on a double-sandwich ELISA, as described above. The equivalent amounts of cardiotoxin in the injected whole venom (0.1 mg/kg, *i.v.* or 0.5 mg/kg, *i.m.*) were estimated to be 0.04 mg/kg or 0.2 mg/kg, respectively, based on a 40% (by dry mass) composition of the whole venom [10]. This additional study aimed to verify if the pharmacokinetics of cardiotoxin when injected alone would be significantly different from that when injected in its native environment (the whole venom).

### Pharmacokinetic analysis

The pharmacokinetic parameters of *N. sumatrana* venom and venom toxins were determined using the method of feathering [28]. The initial phase rate constant (α) and terminal phase rate constant (β) were determined from the slopes of the best-fit lines obtained for the initial phase and terminal phase, respectively, of the log plasma concentration versus time plot. The initial phase half-life (T_1/2α_) and terminal phase half-life (T_1/2 β_) were determined by formula T_1/2α_ or T_1/2β_ = 0.693/α or β. The area under the curve (AUC) was calculated from zero time to the last experimental time point by trapezoidal rule and extrapolated to infinity (AUC_0-∞_) according to the formula: AUC_0-∞_ = AUC_0-t_+C_t_/β, where t is the last experimental time point and C_t_ represents the last serum venom concentration determined at time t.

The distribution rate constants for the transfer between central compartment (designated as 1) and peripheral compartment (designated as 2) were calculated from the equations: k_21_ =  (Aβ + Bα)/(A+B) and k_12_ =  α+ β − k_21_− (αβ/k_21_).

The other important pharmacokinetic parameters were determined as follows:

Systemic clearance, CL  =  dose (F)/AUC_0-∞_

Volume of distribution by area, V_d,area_  =  CL/β

Volume of central compartment, V_c_  =  Dose*_i.v._*/(A + B)

Volume of peripheral compartment, V_p_  =  k_12_/k_21_ (V_c_)

*F_i.v._* is the intravenous bioavailability which is 1.

*F_i.m._* is the intramuscular bioavailability, and was calculated as follows: 
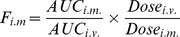



### Statistical analysis

All data are reported as the mean ± S.D. or mean (95% C.I.). Mann-Whitney *U* test was used to compare differences between two independent groups. Kruskal-Wallis H Test is the nonparametric test for the comparison of more than two independent groups. The level of significance was set at *p*<0.05. The statistical analysis was conducted using SPSS 21.0 (SPSS Inc., Chicago, IL, USA).

## Results

### Pharmacokinetics of *N. sumatrana* venom following intravenous administration

**Figure 1:**
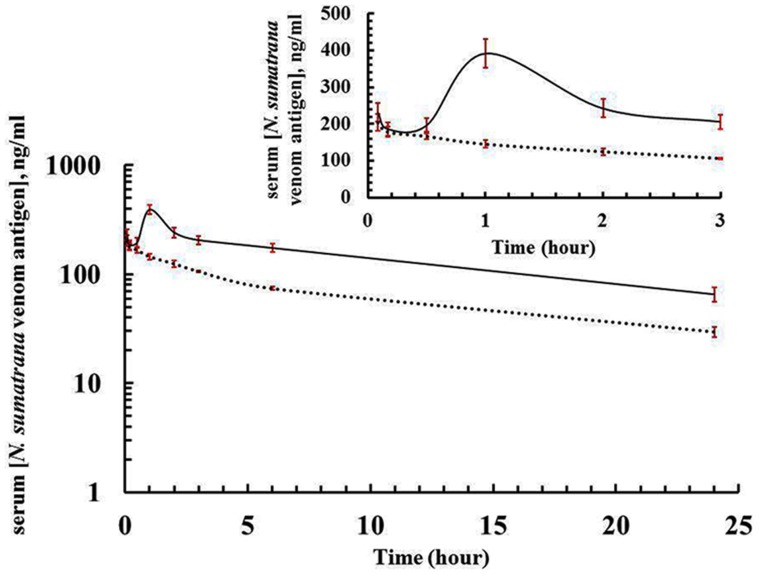
Serum concentration-time profile of *N. sumatrana* venom following intravenous and intramuscular injection of the venom. Rabbits (approximately 2 kg) were injected intravenously (*i.v.*) and intramuscularly (*i.m.*) with a sub-lethal dose of *N. sumatrana* venom. The dose for intravenous injection (dotted line) was 0.1 mg/kg and for intramuscular injection (solid line), 0.5 mg/kg. The serum venom antigen concentrations were determined by double-sandwich ELISA (in semi-logarithmic plot) and given as the means ± S.D. (*n* = 3). The insert shows the serum concentration-time profile of *N. sumatrana* venom following intramuscular injection of venom (in arithmetic plot) during the first 3 h.

The serum concentration-time profiles of whole *N. sumatrana* venom antigen following a single *i.v.* and *i.m.* administrations of venom into rabbits (*n* = 3) are shown in Figure 1.

The *i.v.* serum concentration-time profile of *N. sumatrana* venom (0.1 mg/kg) (Figure 1, dotted line) showed a bi-exponential pattern which was best fitted to a two-compartment model of pharmacokinetics described by the equation C_t_  =  Ae^−αt^ + Be^−βt^: where C_t_ represents the concentration at time, t; A and B represent the venom concentrations at the zero time intercepts of the initial fast phase and terminal slow phase, respectively; while α and β represent the first-order disposition rate constants for the initial fast phase and the terminal phase, respectively.

The venom antigen level declined rapidly within the first 1 h (T_1/2α_ = 0.8 ± 0.3 h) during the initial phase followed by a much slower decline at the terminal phase (T_1/2β_ = 13.6±1.1 h). The volume of distribution by area (V_d,area_) of the venom antigens in rabbits was 1.8±0.03 L, and the systemic clearance (CL) was 91.3±7.8 ml/h, and the AUC_0-∞_ was 2201.2±185.5 ng/ml.h. The distribution rate constant for transfer from central to peripheral compartment (k_12_ = 0.4±0.2 h^−1^) was comparable to that from peripheral to central compartment (k_21_ = 0.5±0.2 h^−1^; *p*>0.05). Consequently, the volume of peripheral compartment (0.8±0.2 L) calculated based on the ratio of k constants was comparable to that of central compartment (1.0±0.1 L).

### Pharmacokinetics of *N. sumatrana* venom following intramuscular administration

The intramuscular administration of whole *N. sumatrana* venom in rabbits yielded a serum concentration-time profile (Figure 1, solid line) with the absorption and distribution phase appeared indistinguishable. The venom antigen level peaked within 1 h at a concentration (C_max_) of 391.7±48.5 ng/ml. The terminal half-life (T_1/2β_ = 12.5±0.9 h), volume of distribution by area (V_d,area_ = 1.7±0.1 L) and the systemic clearance (CL = 94.8±12.7 ml/h) of the venom antigen following *i.m.* injection were not significantly different from that of *i.v.* pharmacokinetic parameters (*p*>0.05) (Table 1).

**Table 1:** Pharmacokinetic parameters of *Naja sumatrana* venom following intravenous and intramuscular administrations of the venom into rabbits.

Parameters	Intravenous (*i.v.*) (LD_50_ = 0.5 µg/g)	Intramuscular (*i.m.*) (LD_50_ = 0.8 µg/g)
A (ng/ml)	101.0±17.4	-
α (h^−1^)	0.9±0.4	-
B (ng/ml)	100.3±1.4	247.7±33.1
β (h^−1^)	0.05±0.004	0.06±0.004
T_1/2 α_ (h)	0.8±0.3	-
T_1/2 β_ (h)	13.6±1.1	12.5±0. 9
C_max_ (ng/ml)	-	391.7±48.5
k_12_ (h^−1^)	0.4±0.2	-
k_21_(h^−1^)	0.5±0.2	-
V_d,area_(L)	1.8±0.03	1.7±0.1
V_c_(L)	1.0±0.1	-
V_p_ (L)	0.8±0.2	-
CL (ml/h)	91.3±7.8	94.8±12.7
AUC_0-∞_ (ng/ml.h)	2201.2±185.5	4617.8±583.8
		(923.6±116.8)*
Bioavailability, *F* (%)	100 (by definition)	41.9±0.2

The dose of *N. sumatrana* venom injected into rabbits (*n* = 3) were *i.v.*: 0.1 mg/kg and *i.m.*: 0.5 mg/kg.

Data were expressed as mean ± S.D. for *n* = 3.

* AUC_0-∞_ value was adjusted to the dosage of *i.v.* injection, *i.e.* 0.1 mg/kg.

The AUC_0-∞_ of *N. sumatrana* venom when injected intramuscularly (0.5 mg/kg) was 4617.8±583.8 ng/ml.h. However, when adjusted to the intravenous venom dose (0.1 mg/kg), the normalized AUC_0-∞_ of the venom antigens following *i.m.* administration was 923.6±116.8 ng/ml.h, which was significantly lower than the *i.v.* AUC_0-∞_ value (2201.2±185.5 ng/ml.h; *p*<0.05). The *i.m.* bioavailability (*F_i.m._*) calculated from the two AUC_0-∞_ values were 41.9±0.2%.

### Immunological cross-reactions of *N. sumatrana* venom toxins (phospholipase A_2_, neurotoxin and cardiotoxin)

The phospholipase A_2_, neurotoxin and cardiotoxin were isolated and purified from *N. sumatrana* venom. The protein identity of each toxin was confirmed by MALDI-TOF/TOF and is shown in Table 2.

**Table 2:** MALDI-TOF/TOF identification of phospholipase A_2_, neurotoxin and cardiotoxin isolated from *Naja sumatrana* venom.

Venom toxins	Matched peptide sequences	Accession No. and Protein Family	Protein Score	% coverage
Phospholipase A_2_	SWWHFADYGCAYCGR	Q92084	527	51
	GGSGTPVDDLDR	Neutral Phospholipase A_2_- A		
	CCQIHDNCYNEAEK			
	CWPYFK			
	TYSYECSQGTLTCK			
	GGNNACAAAVCDCDR			
Neurotoxin	LECHDQQSSQTPTTTGCSGG	Q9PSN6	82	74
	ETNCYK	Short neurotoxin		
	NGIEINCCTTDR			
Cardiotoxin	LVPLFYK	P60302	233	39
	MFMVATPK	Cardiotoxin 3		
	RGCIDVCPK			
	GCIDVCPK			
	YVCCNTDR			

Protein scores greater than 67 are significant (*p*<0.05).

The mass spectra acquired were searched against all non-redundant NCBI protein database with taxonomy set to Serpentes (taxid: 8570).

**Figure 2:**
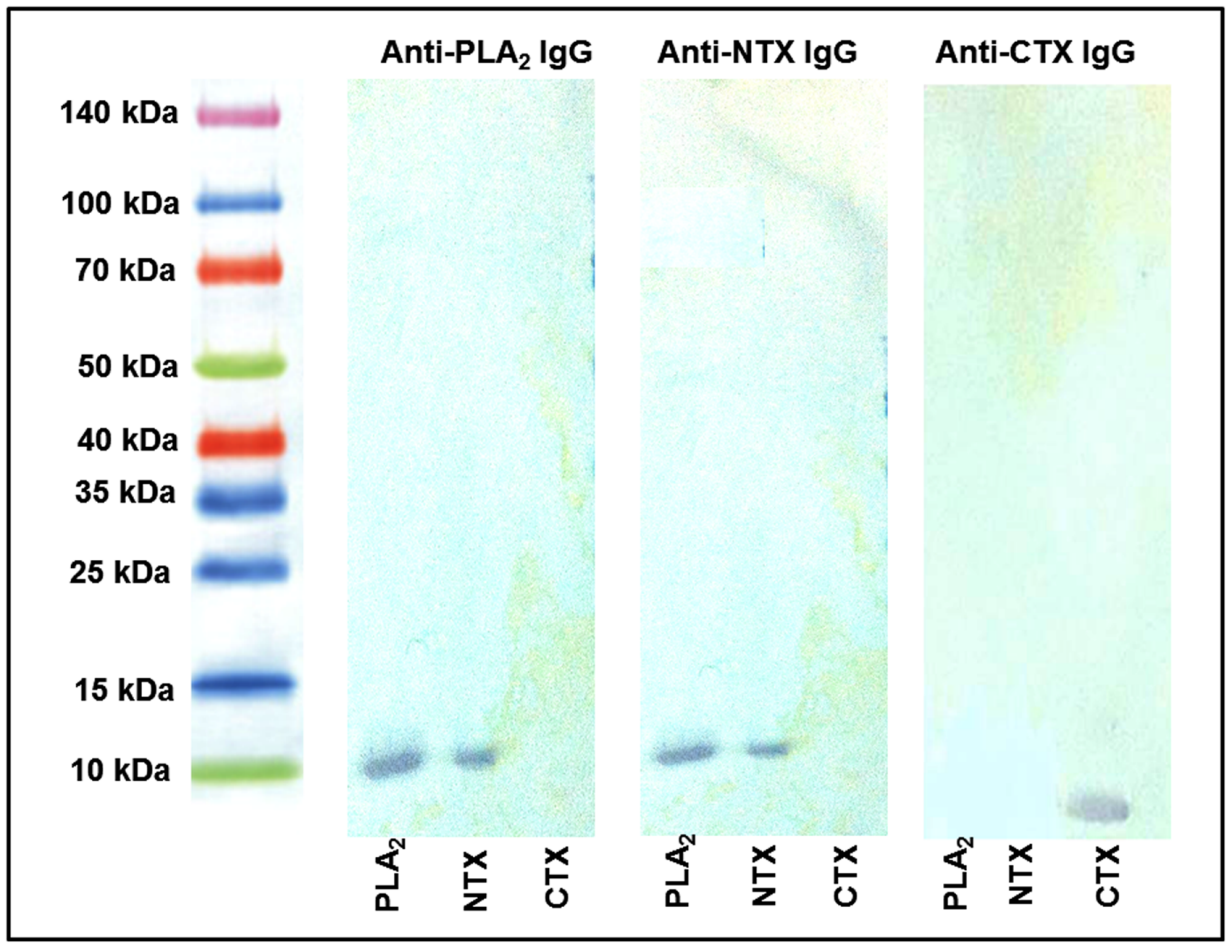
Immunological cross reactions between *N. sumatrana* venom toxins as analyzed by immunoblotting. Venom toxins (10 µg each of phospholipase A_2_, neurotoxin and cardiotoxin) was electrophoresed on a SDS-PAGE gel (15%, reducing condition), and electro-transferred to a PVDF membrane. This was followed by subsequent incubation with primary antibody (anti-PLA_2_ IgG, anti-NTX IgG and anti-CTX IgG (dilution of 1: 500) and goat anti-rabbit IgG-HRP (dilution of 1∶1000). Substrate solution (Novex HRP Chromogenic Substrate (TMB), Invitrogen) was added for colorimetric development.

Indirect ELISA and double-sandwich ELISA demonstrated extensive cross-reactions between phospholipase A_2_ and neurotoxin (>50%), but not between these two toxins and cardiotoxin (Table 3). These findings were supported by Western blot results (Figure 2): the anti-PLA_2_ IgG only reacted with the phospholipase A_2_ and neurotoxin, but not with cardiotoxin; and similarly, the anti-NTX IgG only reacted with the neurotoxin and phospholipase A_2_, but not with cardiotoxin. Anti-CTX IgG reacted only with cardiotoxin, but neither with phospholipase A_2_ nor neurotoxin. The neurotoxin appears to migrate to a higher position than it should (*i.e.* in the same position as phospholipase A_2_) (Figure 2). To further examine this phenomenon, we performed protein mass analysis and confirmed that the neurotoxin isolated is indeed short neurotoxin with a molecular mass of 6.5 kDa (unpublished data). The mass increase of neurotoxin as observed from SDS-PAGE could be attributed to the oxidation of Trp or Met residues in the neurotoxin [29]. Similar observation of abnormally high molecular mass neurotoxin has also been reported from *Ophiophagus hannah* venom [30].

**Table 3:** Immunological cross-reactivity of *Naja sumatrana* venom toxins by indirect ELISA and double-sandwich ELISA.

INDIRECT ELISA		DOUBLE SANDWICH ELISA	
Venom Toxins	% cross-reactivity	Venom Toxins	% cross-reactivity
anti-PLA_2_ IgG		anti-PLA_2_ IgG	
PLA_2_	100	PLA_2_	100
NTX	155.9±26.7	NTX	59.4±13.4
CTX	0.8±0.3	CTX	0
anti-NTX IgG		anti-NTX IgG	
PLA_2_	55.1±2.2	PLA_2_	72.5±10.1
NTX	100	NTX	100
CTX	0.8±0.2	CTX	0.7±0.3
anti-CTX IgG		anti-CTX IgG	
PLA_2_	0	PLA_2_	0
NTX	3.2±0.4	NTX	0
CTX	100	CTX	100

For indirect ELISA, the immunoplate was coated with 100 ng/ml of the venom toxin as antigen, and reacted with anti-PLA_2_IgG, anti-NTX IgG and anti-CTX IgG (dilution of 1∶200), respectively. For double-sandwich ELISA, the immunoplate was coated with 4 µg/ml of anti-PLA_2_ IgG, anti-NTX IgG and anti-CTX IgG respectively; and subsequently incubated with 100 ng/ml of venom toxin.

The venom toxins used were phospholipase A_2_ (PLA_2_), neurotoxin (NTX) and cardiotoxin (CTX). Data were expressed as mean ± S.D. for *n* = 3.

### Pharmacokinetics of major toxins following intramuscular and intravenous administration

**Figure 3:**
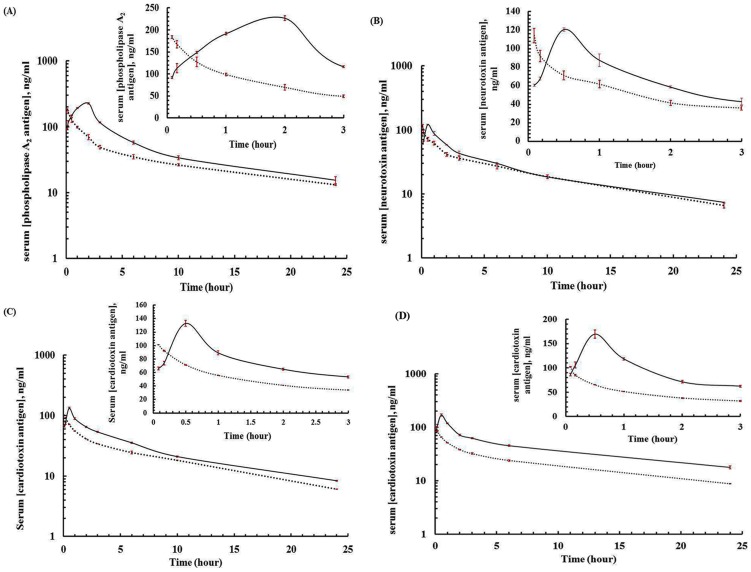
Serum concentration-time profiles of *N. sumatrana* venom toxins. The serum toxin-antigen concentrations of each individual toxin following intravenous (*i.v.*) (dotted line) and intramuscular (*i.m.*) (solid line) injection of *N. sumatrana* venom phospholipase A_2_ (3A), neurotoxin (3B) and cardiotoxin (3C). Figure 3D shows the serum cardiotoxin antigen concentrations following injection of the whole *N. sumatrana* venom. Toxin concentrations were determined by respective double-sandwich ELISA. Data are given as means ± S.D. (*n* = 3). The insert shows serum concentration-time profile (in arithmetic plot) during the first 3 h.

The serum concentration-time profiles of purified *N. sumatrana* venom phospholipase A_2_, neurotoxin and cardiotoxin following single *i.v.* or *i.m.* administrations into rabbits (*n* = 3) are shown in Figure 3A–C. All of the intravenous profiles showed a bi-exponential pattern which was best fitted to a two-compartment pharmacokinetic model represented by the following equation: C_t_  =  Ae^−αt^ + Be^−βt^. The antigen concentrations in general decreased rapidly within a distribution half-life (T_1/2α_) of 0.5–0.7 h during the initial phase and followed by a declining terminal phase with half-life (T_1/2β_) of 8–12 h. On intramuscular routes, it contrast to the multiple peaks (C_max_) in the case of whole venom, we observed a single peak for toxin absorption at 0.5 h (T_max_ for neurotoxin and cardiotoxin) or 2 h (T_max_ for phospholipase A_2_) (Figure 3A–C, solid line). The intramuscular profile subsequently followed that of intravenous profile with a linear declining curve, illustrating the terminal phase of the serum concentration-time course.

The *i.v.* and *i.m.* pharmacokinetic parameters of all three major toxins were shown in Table 4. Most of the key *i.m.* pharmacokinetic parameters of the toxins (especially related to distribution and elimination processes) were not significantly different from the corresponding *i.v.* pharmacokinetic parameters (*p*>0.05). The intramuscular bioavailability (*F_i.m._*) of the toxins were estimated by comparing the dose-adjusted intramuscular AUC_0-∞_ of toxin to the corresponding intravenous AUC_0-∞_.

**Table 4:** Pharmacokinetic parameters of *Naja sumatrana* venom toxins (phospholipase A_2_, neurotoxin and cardiotoxin) following intravenous and intramuscular administration of the venom toxins into rabbits.

Venom toxins	Phospholipase A_2_		Neurotoxin		Cardiotoxin	
Parameters	Intravenous (*i.v.* 0.05 mg/kg)	Intramuscular (*i.m.* 0.1 mg/kg)	Intravenous (*i.v.* 0.05 mg/kg)	Intramuscular (*i.m.* 0.07 mg/kg)	Intravenous (*i.v.* 0.05 mg/kg)	Intramuscular (*i.m.* 0.15 mg/kg)
A (ng/ml)	141.7±9.6	-	67.5±6.2	-	63.6±4.2	-
α (h^−1^)	1.1±0.1	-	1.4±0.3	-	1.3±0.2	-
B (ng/ml)	52.6±3.9	77.7±5.1	44.0±5.4	48.8±4.2	41.4±1.3	59.9±2.2
β (h^−1^)	0.06±0.004	0.07±0.008	0.08±0.008	0.08±0.005	0.08±0.001	0.09±0.001
T_1/2 α_ (h)	0.7±0.03	-	0.5±0.1	-	0. 6±0.1	-
T_1/2 β_(h)	11.7±0.8	10.18±1.18	8.8±0.9	8.6±0.5	8.6±0.1	8.2±0.1
C_max_ (ng/ml)		226.6±5.5		120.7±2.4		133.0±5.7
k_12_ (h^−1^)	0.6±0.02	-	0.7±0.1	-	0.6±0.1	-
k_21_(h^−1^)	0.3±0.04	-	0.6±0.1	-	0.6±0.1	-
V_d,area_ (L)	1.6±0.03	1.4±0.2	2.1±0.3	2.2±0.2	2.2±0.1	2.0±0.03
V_c_(L)	0.5±0.02	-	0.9±0.1	-	1.0±0.1	-
V_p_(L)	0.9±0.1	-	1.0±0.1	-	1.1±0.02	-
CL(ml/h)	95.8±4.8	95.8±4.8	164.1±11.6	164.1±11.6	173.7±2.1	173.7±0.001
AUC_0-∞_ (ng/ml.h)	1045.4±52.6	1499.9±154.0	611.4±43.3	694.9±2.4	575.7±7.0	788.3±23.9
		(750.0±77.0)*		(496.2±1.7)*		(262.7±8.0)*
Bioavailability, *F* (%)	100	68.6±0.8	100	81.5±0.6	100	45.6±0.1

Data were expressed as mean ± S.D. for *n* = 3. * AUC_0-∞_ values were adjusted to the dosage of *i.v.* injection, *i.e.* 0.05 mg/kg for phospholipase A_2_, neurotoxin and cardiotoxin.

### Pharmacokinetics of cardiotoxin following intravenous or intramuscular administrations of whole *N. sumatrana* venom

The dotted-line curve in Figure 3D shows the serum cardiotoxin concentration following intravenous whole venom administration that declined in a bi-exponential manner; while the solid-line curve depicts its intramuscular absorption with a T_max_ of 0.5 h and a terminal phase parallel to that of intravenous profile. The pharmacokinetic parameters of the “*in-venom*” cardiotoxin following the intravenous and intramuscular administrations are shown in Table 5. The pharmacokinetic parameters of cardiotoxin when only the toxin was administered are also listed for comparison (see Discussion). Most of the pharmacokinetic parameters of the *in-venom* cardiotoxin were comparable with values obtained when only purified cardiotoxin was administered, with the major exceptions of a longer elimination half-life (T_1/2β_) and a lower clearance (CL) for the *in-venom* cardiotoxin (Table 5).

**Table 5:** Pharmacokinetics parameters of cardiotoxin following intravenous and intramuscular administration of whole *Naja sumatrana* venom into rabbits.

Parameters	Intravenous		Intramuscular	
	Injection of whole venom	Injection of cardiotoxin	Injection of whole venom	Injection of cardiotoxin
A(ng/ml)	61.3±1.2	63.6±4.2	-	-
α (h^−1^)	1.4±0.03	1.3±0.2	-	-
B (ng/ml)	39.1±1.2	41.4±1.3	73.7±3.0	59.9±2.2
β (h^−1^)	0.06±0.001	0.08±0.001	0.1±0.004	0.09±0.001
T_1/2α (_h)	0.5±0.01	0.6±0.1	-	-
T_1/2 β_(h)	11.0±0.2	8.6±0.1	11.6±0.9	8.2±0.1
C_max_ (ng/ml)			169.4±8. 5	133.0±5.7
k_12_ (h^−1^)	0.8±0.01	0.6±0.1	-	-
k_21_(h^−1^)	0.6±0.02	0.5±0.1	-	-
V_d,area_(L)	1.9±0.1	2.2±0.1	2.0±0.2	2.0±0.03
V_c_(L)	0.8±0.01	1.0±0.1	-	-
V_p_(L)	1.0±0.04	1.1±0.02	-	-
CL (ml/h)	119.8±2.4	173.7±2.1	121.7±0.2	173.7±0.001
AUC_0-∞_ (ng/ml.h)	668.0±13.0	575.7±7.0	1320.1±35.6	788.3±23.9
			(264.0±7.1)*	(262.7±8.0)*
Bioavailability, *F* (%)	100	100	39.5±1.1	45.6±0.1

The sub-lethal dose of *N. sumatrana* venom injected into rabbits (*n* = 3, approximately 2 kg each) were *i.v.*: 0.1 mg/kg and *i.m.*: 0.5 mg/kg.

Data were expressed as mean ± S.D. for *n* = 3. The pharmacokinetic parameters when only cardiotoxin was injected are taken from Table 4.

* AUC_0-∞_ values were adjusted to the dosage of *i.v.* injection, *i.e.* 0.1 mg/kg.

## Discussion

Generally, the serum concentration-time profile of the venom/toxin injected intravenously can be described by an open two-compartment pharmacokinetic model where the venom or toxin is distributed between the central and peripheral compartments. The distribution half-life of *N. sumatrana* venom (T_1/2α_ = 0.77 h) is comparable to the value obtained for *N. sputatrix* venom in an earlier study (T_1/2α_ = 0.5 h) [22], and to that for the African cobra venoms (T_1/2α_ = 22.2–30.5 min) [15] although a three–compartment pharmacokinetic model was applied in the latter case. The terminal half-life (T_1/2β_ = 13.6 h) of *N. sumatrana* venom was not significantly different to that of *N. sputatrix* venom (T_1/2β_ = 15.4 h) [22], indicating that the elimination of the venom antigens of these two Southeast Asian spitting cobras occurred at similar rate.

The volume of central compartment approximated 1 L for *N. sumatrana* venom, indicating that on intravenous administration, the venom distributes rapidly and uniformly not only in the plasma of the animal (28–50 ml/kg for rabbit) but also in highly perfused tissues and interstitial fluids as well in view of its major content being water-soluble low molecular mass toxins (<10 kDa) which may readily permeate the vascular endothelium [10]. As the ratio of the inter-compartmental transfer rate constants k_12_ and k_21_ for the venom approximates unity (k_12_/k_21_ = 0.78), this means that at equilibrium (when inter-compartmental transfer rates are equal), the amount of venom antigens in both the compartments do not vary significantly. This finding corroborates with that obtained in our earlier pharmacokinetic study of *N. sputatrix* venom (k_12_/k_21_ = 1) [22]. On the other hand, the large V_d,area_ of the venom (1.8 L, more than 10-fold of the total blood volume of a 2-kg rabbit) suggests that this cobra venom antigens distribute extensively to the peripheral or extra-vascular tissues. This seems to be a general phenomenon for venom antigens distribution in experimentally envenomed animals [12], [13], [15]. Venom toxins that are distributed widely into the peripheral compartment may be associated with the rebound phenomenon that sometimes occur during antivenom therapy as rapid immunodepletion of venom toxins in the blood favors the redistribution of venom antigens from the peripheral back into the central compartment [31], [32].

To study the absorption of venom from the non-vascular parenteral administration site, the pharmacokinetics of *N. sumatrana* venom was studied following an intramuscular administration of the venom (sub-lethal dose) into rabbits. The resulting serum venom antigen concentration-time profile showed a relatively fast absorption of some venom antigens within the first few minutes. A subsequent short lag observed during the absorption phase of the venom was probably due to the absorption of some venom antigens via the lymphatic route from the injection site into blood circulation [20]. However, the serum concentration-time profile of the intramuscularly injected whole venom yields apparently indistinguishable absorption and distribution phases of the various toxins. The indistinguishable absorption and distribution phase reflects the continuous absorption of various antigenic venom components that occur simultaneously with their respective distribution phases. There was only one major serum concentration peak seen at 1 h, presumably caused by the summated absorption of a bulk of phospholipase A_2_ (T_max_∼2 h) and the three-finger toxins, *i.e.* neurotoxin and cardiotoxin (T_max_∼½ h) into the systemic circulation occurring at a rate in close proximity with each other. Rapid absorption of the venom with a short T_max_ correlates with the fast onset of neurotoxic effect in cobra envenoming, where the venom is known to exert direct inhibitory action on the neuromuscular junction via a postsynaptic blockade [33]. Administration of the venom by intramuscular route did not alter T_1/2β_, V_d,area_ and CL of the venom antigens. This indicates that the elimination (and not the absorption) process is likely the rate-limiting step for the terminal phase of the pharmacokinetic profile of intramuscularly injected venom.

As demonstrated from ion-exchange HPLC studies [10], *N. sumatrana* venom contains more neurotoxin and cardiotoxin (17% and 40%, respectively) compared to *N. sputatrix* venom (consists of 8% neurotoxin and 35% cardiotoxin). However, *N. sputatrix* venom has substantially greater amount (35%) of phospholipase A_2_ than *N. sumatrana* venom (28%). This may account for the somewhat greater plasma clearance of *N. sumatrana* venom (91.3 ml/h) compared to that of *N. sputatrix* venom (68.7 ml/h), since the smaller three-finger toxins (more abundant in *N. sumatrana* venom) are cleared faster than the larger phospholipase A_2_ (more abundant in *N. sputatrix* venom), especially via the renal excretion route.

The bioavailability of *N. sumatrana* venom following *i.m.* injection was 41.9%, indicating incomplete absorption of the antigenic venom components from the injection site into the systemic circulation. This may be due to strong affinities of the cobra venom toxins at the injection site [12], [21], and this hypothesis correlates well with cobra venom's prominent toxic effect on local tissues that lasts for days to weeks [9], [34]–[36]. The bioavailability of *N. sumatrana* venom (*F_i.m._*) was similar to that of *N. sputatrix* venom (*F_i.m_* = 41.7%), which is a reflection of the fact that the cardiotoxin represents the bulk of both venoms (*F_i.m_.* of cardiotoxin was 46%, discussed below).

### Pharmacokinetics of the individual major toxins of *N. sumatrana* venom

Since snake venom is a mixture of hundreds of proteins and peptides, it is therefore virtually impossible to investigate the pharmacokinetics of each individual toxin when the whole venom was administered into rabbits. As such, in this study, we only selected three representative toxins of *N. sumatrana* venom (neurotoxin, cardiotoxin and phospholipase A_2_) for pharmacokinetic investigations. These three toxins also represent the major types of toxins in the venom.

It should be noted that accurate quantitative measurement of individual toxins in the serum of experimentally envenomed animal using ELISA assay is not always feasible because of the immunological cross-reactivities observed among the snake venom toxins [37]. Indeed, our immunological cross-reaction studies revealed extensive cross-reactivity between the phospholipase A_2_ and polypeptide neurotoxin purified from *N. sumatrana* venom, demonstrating that unrelated venom proteins of distinctive primary structures and biological functions may share common antigenic domains [27], [38]. As such, in the present report the pharmacokinetics of *N. sumatrana* venom purified phospholipase A_2_, neurotoxin and cardiotoxin was studied after intravenous or intramuscular injection of a sub-lethal dose of each toxin into rabbits. Double-sandwich ELISA was developed in which specific anti-toxin IgG's (*i.e.* anti-PLA_2_ IgG, anti-NTX IgG, anti-CTX IgG) were used to measure the serum toxin antigen levels following injections of the individual toxins into rabbits.

The individual serum concentration-time profiles of the toxins, as with the whole venom, injected intravenously were also best fitted to an open two-compartment pharmacokinetic model, where the toxins distributed between central and peripheral compartments. Following intravenous administration, the individual toxins *i.e.* phospholipase A_2_, neurotoxin and cardiotoxin demonstrated shorter distribution half-lives (0.56–0.66 h) compared to the whole venom (0.93 h), reflecting a more rapid distribution of the purified toxins on entering the systemic circulation.

On the other hand, unlike that observed for the whole venom, there was no fluctuation pattern during the absorption and/or distribution phase in the serum concentration-time profile of individual toxins administered intramuscularly. The significant differences in the absorption of the whole venom and toxins were also reflected by the time to reach peak concentration (T_max_). The neurotoxin and cardiotoxin antigens reached their respective peak concentrations much faster than phospholipase A_2_, indicating fast absorption of these two low molecular mass toxin molecules (approx. 8 kDa) from the injection site into the systemic circulation. These principal cobra toxins are known to directly target receptors and cellular membranes, inducing rather rapid tissue responses compared to some viperid toxins the actions of which involve intermediate steps to accomplish the toxic effect, for instance, coagulopathy secondary to defribrinogenation induced by thrombin-like enzymes [32]. The fast absorption of neurotoxin and cardiotoxin likely accounts for the rapid onset of the systemic effects upon cobra envenomation *i.e*. neuromuscular paralysis and cardiac complications [7], [9], [39]. In view of the rapid absorption of these major toxins, meticulous monitoring for early institution of antivenom when indicated becomes crucial in order to alleviate the severity of syndrome and to preempt fatal outcome.

Furthermore, all the three toxins exhibited a large V_d,area_ (1.6–2.2 L) which are >10 fold of the total blood volume of a rabbit, suggesting that the toxin antigens distributed extensively into the peripheral tissues. This finding is congruent with the large volume of distribution of the whole venom in rabbits as described above. Both the neurotoxin and cardiotoxin (2.0–2.2 L) showed a larger V_d,area_ compared to the phospholipase A_2_ (1.4 L), and this may be because low molecular mass proteins like neurotoxin and cardiotoxin (with molecular mass of approximately 7–8 kDa) cross the capillary endothelium more easily than do the larger proteins [13] such as phospholipase A_2_ (16 kDa).

In this study, the terminal half-lives (T_1/2β_) of neurotoxin and cardiotoxin were similar (8.6–8.8 h) but shorter than that of phospholipases A_2_ (11.7 h). This finding is consistently reflected in the systemic clearance of the three toxins, where the clearance values of neurotoxin and cardiotoxin were significantly larger (indicative of faster elimination) than that of phospholipase A_2_. Assuming that the elimination takes place primarily from the central compartment and probably via the renal excretion route, the faster clearance of neurotoxin and cardiotoxin can be explained by the higher vascular permeability of the two toxins as both are low molecular mass peptides. However, the T_1/2β_ values for the neurotoxin and cardiotoxin determined in this study are substantially different from the terminal half-lives of African cobras' α-neurotoxin (15–29 h, in rabbits) [15] and that of cytotoxin from Chinese cobra, *Naja naja atra* (3.5 h, in rabbits) [12], suggesting intrageneric variations in the pharmacokinetics of these cobra three-finger toxins.

Among the three major toxins, *N. sumatrana* neurotoxin has the most complete systemic absorption from the injection site, as evidenced by its higher intramuscular bioavailability (*F_i.m._* = 81.5%) than that of phospholipase A_2_ (68.6%) and cardiotoxin (45.6%). This is in agreement with the finding of Ismail *et al.* (1998) [16], who reported a bioavailability of 88% for *Walterinnesia aegyptia* α-neurotoxin. Interestingly, the *F_i.m_*_._ of cardiotoxin was only 45.6%, presumably due to the strong binding affinity of cardiotoxin to the tissues at the injection site resulting in a poor absorption of cardiotoxin into the systemic circulation. On the other hand, the *F_i.m_*_._ of the phospholipase A_2_ was 68.6%, indicating that a substantial amount of the toxin remained at the injection site. Indeed, bites from *N. sumatrana* (and most *Naja* cobras) can produce local envenomation characterized by local tissue necrosis involving the cutaneous, muscular and connective tissue layers [2], [9], [34], [40], [41]. Cardiotoxin and phospholipase A_2_ have been reported to interact synergistically and possess potent cytolytic activity [42], [43], and their substantial unabsorbed amount at the injection site seem to suggest that their toxic effects play an important role in local envenoming, which consequences include tissue necrosis following cobra bites, as well as venom ophthalmia in venom-spitted victims [44].

Although the *i.v.* pharmacokinetic behavior of neurotoxin is similar to that of cardiotoxin (particularly in having a rapid absorption with a short T_max_), their intramuscular bioavailabilities differed markedly. The relatively low bioavailability of cardiotoxin would suggest that the systemic effects of cardiotoxin may not be that prominent in cobra envenomation, even though the venom contains relatively large amount of cardiotoxins (40% of venom content [10]). Furthermore, the neurotoxin is known to be much more lethal than both the cardiotoxin and phospholipases A_2_, with an approximate 10-fold lower LD_50_ in mice (0.1 µg/g, [34]). It belongs to α-neurotoxins with high intrinsic activity of inhibiting the motor endplate nicotinic receptors vis-à-vis that of cardiotoxins and phospolipases A_2_, the target receptors of which are primarily different and their actions are not crucial in mediating neuromuscular paralysis - the central cobra envenoming feature that leads to rapid death [34]. This is consistent with clinical reports where rapid onset of neuromuscular paralysis (caused by neurotoxins) is the most common fatal manifestation of systemic cobra envenomation, where victims may succumb to respiratory failure and death ensues within minutes to hours [9], [36], [45]. The pharmacokinetic result in addition to the neuromuscular blockade activity of neurotoxin generally supports the hypothesis that the neurotoxin plays the principal role in systemic envenomation of *N. sumatrana*, and should be one of the most crucial toxins to be targeted by antivenom. Nevertheless, variations of neurotoxins across cobra species have been reported on their structures and activities, and the phenomenon is likely the clue to varied efficacies of commercially available antivenoms in the cross-neutralization of cobra venoms in the region [46]. The pharmacokinetic profiling method hence appears useful in validating the toxin's role from the pharmacokinetic aspect, and may be further utilized as a tool in assessing antivenom efficacy on the targeted toxin derived from different cobras.

### Pharmacokinetics of cardiotoxin following intravenous or intramuscular administrations of whole *N. sumatrana* venom

In view of the negligible immunological cross-reactivity between cardiotoxin with phospholipase A_2_ and neurotoxin, it is possible to accurately determine the serum concentration of cardiotoxin following intravenous or intramuscular administration of the whole *N. sumatrana* venom using the same double-sandwich ELISA developed. This study would help to shed light on whether the pharmacokinetics of an individual toxin could be altered by other venom constituents, and whether the information gathered from the pharmacokinetic study of individual toxins can be applied in situations where the whole venom was injected.

The serum concentration-time profile of cardiotoxin when whole venom was injected was found to be similar to that when only purified cardiotoxin was injected (Figure 3C and 3D). It is however noted that when whole venom was injected, cardiotoxin exhibited a longer T_1/2β_ and a lower CL than when only cardiotoxin was injected. The results therefore suggest that the rate of elimination of cardiotoxin in the whole venom is likely affected by the presence of other venom components in the venom due to competition among various venom components for the elimination processes. The results reflect that in *N. sumatrana* envenomation, pharmacokinetic characteristics of individual major toxins can be largely applied to situations where the whole venom is injected, with the possible exception that the rate of elimination of the toxins determined may be higher than that of the whole venom. On the other hand, the intramuscular bioavailability (*F_i.m_*) of cardiotoxin injected with whole venom (39.5%) was similarly low, if not even lower, compared to the *F_i.m._* of cardiotoxin when only the toxin was administered (45.6%), consistent with the indication that cardiotoxin remained substantially unabsorbed at the injection tissue site.

### Conclusions

In general, the elimination half-life of the whole venom is determined by the toxic components with the longest T_1/2β_ (phospholipase A_2_ in the case of *N. sumatrana* venom), while its intramuscular bioavailability is influenced more by the toxic components that is present most abundantly in the venom (cardiotoxin, in this case). In the present pharmacokinetic study of *N. sumatrana* venom and toxins, both the neurotoxin and cardiotoxin were rapidly absorbed intramuscularly in the rabbits, with neurotoxin achieving the highest systemic bioavailability, while the cardiotoxin and phospholipase A_2_ possess relatively lower bioavailabilities. These pharmacokinetic findings therefore suggest that the neurotoxin is the principal toxin in systemic envenomation (fatal neuromuscular paralysis), while significant amounts of the cardiotoxin and phospholipase A_2_ remain bound to the injection site, causing local tissue destruction. Using toxin-specific ELISA, the study also shows that the cobra venom pharmacokinetics is likely an approximation of the pharmacokinetics of individual toxins except for parameters relating to elimination rate due to possible competition of various toxins for the process *in vivo*.
