# Correction: Pharmacokinetics of *Naja sumatrana* (Equatorial Spitting Cobra) Venom and Its Major Toxins in Experimentally Envenomed Rabbits

**DOI:** 10.1371/journal.pntd.0003277

**Published:** 2014-09-30

**Authors:** The PLOS Neglected Tropical Diseases Staff

Figure 2 is incorrect. A different file was inadvertently uploaded by the authors upon submission of the revised article. The authors have provided a corrected version here.

**Figure 2:**
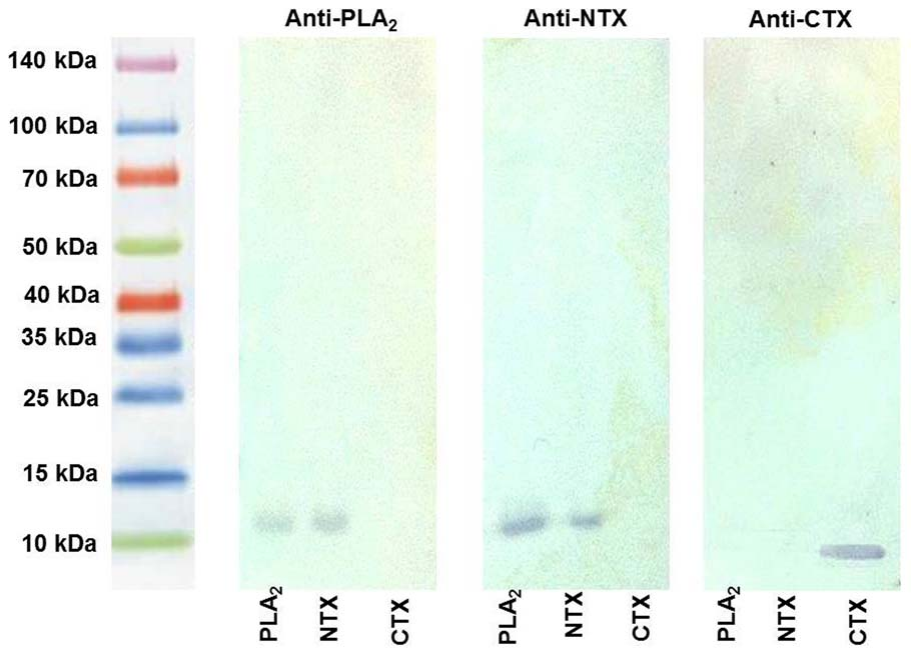
Immunological cross reactions between N. sumatrana venom toxins as analyzed by immunoblotting. Venom toxins (10 µg each of phospholipase A2, neurotoxin and cardiotoxin) was electrophoresed on a SDS-PAGE gel (15%, reducing condition), and electro-transferred to a PVDF membrane. This was followed by subsequent incubation with primary antibody (anti-PLA2 IgG, anti-NTX IgG and anti-CTX IgG (dilution of 1: 500) and goat anti-rabbit IgG-HRP (dilution of 1:1000). Substrate solution (Novex HRP Chromogenic Substrate (TMB), Invitrogen) was added for colorimetric development.
